# Comprehensive Characterization of Chemical Composition and Antioxidant Activity of Lignan-Rich Coniferous Knotwood Extractives

**DOI:** 10.3390/antiox11122338

**Published:** 2022-11-25

**Authors:** Nikolay V. Ul’yanovskii, Aleksandra A. Onuchina, Anna V. Faleva, Natalia S. Gorbova, Dmitry S. Kosyakov

**Affiliations:** 1 Laboratory of Natural Product Chemistry and Bioanalytics, Core Facility Center “Arktika”, Northern (Arctic) Federal University, 163002 Arkhangelsk, Russia; 2 Federal Center for Integrated Arctic Research, 163000 Arkhangelsk, Russia

**Keywords:** lignans, coniferous knotwood, extractives, antioxidant activity, non-target screening

## Abstract

A knotwood of coniferous trees containing large amounts of polyphenolic extractives is considered a promising industrial-scale source of lignans possessing antioxidant properties and other bioactivities. The present study is aimed at a detailed characterization of the chemical composition and antioxidant activity of lignan-rich extractives obtained from the knotwood of the Norway spruce, Scotch pine, Siberian fir, and Siberian larch growing in the European North of Russia as a region with a highly developed forest industry. To achieve this, a comprehensive approach based on a combination of two-dimensional NMR spectroscopy with high-performance liquid chromatography—high-resolution Orbitrap mass spectrometry, and the determination of antioxidant activity by the three complementary methods were proposed. The studied knotwood samples contained from 3.9 to 17% of extractive substances and were comparable to Trolox’s antioxidant activity in the single-electron transfer processes and superoxide radical scavenging, which is associated with the predominance of polyphenolic compounds. The latter was represented by 12 tentatively identified monolignans and 27 oligolignans containing 3–5 phenylpropane units in their structure. The extracts were characterized by an identical set of lignans and differed only in the ratios of their individual compounds. Other components of the knotwood were flavonoids taxifolin, quercetin (Siberian larch), and three stilbenes (pinosylvin, its methyl ester, and pterostilbene), which were identified in the Scotch pine extractives. Sesquiterpene juvabione and its derivatives were found in extracts of Siberian larch knotwood.

## 1. Introduction

Lignans make up an extensive group (with more than a thousand representatives) of secondary plant metabolites that are widely distributed in the plant kingdom [1,2,3]. They are mainly dimeric (in some cases trimeric and tetrameric) lignin-related polyphenolic compounds of eight different types, formed from phenylpropane structural units (usually hydroxycinnamic acids and alcohols) and possessing high antioxidant activity [4]. The biological role of lignans in plant tissues is still not entirely clear; however, it is usually associated mainly with the defense against external agents [5,6].

Due to the unique biological activity involving, along with pronounced antioxidant properties, antitumor, hepatoprotective and cardioprotective effects, which play an important role in the metabolism of humans and animals (transformation into mammalian enterolignans by the gut microbiota), lignans are considered to be promising pharmacological and nutraceutical agents [7,8]. The studies of lignans and their natural sources have rapidly developed in recent decades. This can be clearly illustrated in the review paper by Pan et al. [9], who reported the discovery, identification, and characterization of more than 250 new lignans over five years.

The most important food sources of lignans are Chinese lemongrass (*Schisandra chinensis*) fruits (content up to 2%), flax and sesame seeds (~0.3%), and cereals (~0.001%) [10]. Their biomass, with the exception of cereals, is also used for the industrial production of lignans. At the same time, there is an alternative widely available and relatively recently discovered [11,12] source of lignans which fully meets the needs of the pharmaceutical industry and other applications–the compression wood (knots and roots) of coniferous trees. The high mechanical loads experienced by the knot and root tissues inside the tree trunk initiate the biosynthesis of lignans as a response to mechanical stress [13,14]. The coniferous knotwood, which constitutes a significant part of wood waste in the timber industry, is especially rich in lignans. According to Willför et al. [13], the content of lignans in the Norway spruce (*Picea abies*) knotwood varies in the range of 6–24%, while at least two-thirds of the total amount is accounted for by hydroxymatairesinol known as the enterolactone precursor with anticancer activities [15,16]. Other species of coniferous trees contain somewhat lower amounts of lignans in their knotwood and may differ in their dominant compound: secoisolariciresinol (Siberian larch, Siberian fir, Silver fir, and Douglas fir) and nortrachelogenin (*Scotch pine*) [11,17,18,19]. The less abundant components of the lignan fraction were tentatively identified as conidendrin, liovil, matairesinol, pinoresinol, lariciresinol, and isolariciresinol [13,17,19,20,21]. In addition to monomeric aglycons, glycosylated derivatives, ethers with guaiacol, and oligolignans with different degrees of polymerization were also found as minor constituents of knotwood extracts [21,22,23].

Willför et al. [20] reported that, due to the high content of lignans, coniferous knotwood extracts possess a pronounced ability to inhibit lipid peroxidation and can be several times superior to Trolox in this parameter. The same pattern was observed for the scavenging of superoxide and peroxyl radicals in vitro. Among the individual lignans, hydroxymatairesinol had the highest superoxide radical scavenging activity, while the results of comparing the lignans in lipid peroxidation tests were inconsistent. Using an independent amperometric method (electrochemical oxidation on a glassy carbon electrode), Yashin et al. [24] confirmed the high antioxidant activity of lignans isolated from larch wood and ranked them according to this parameter in the following sequence: secoisolariciresinol~isolariciresinol < lariciresinol. It is worth noting that most of the studied lignans were superior in their antioxidant activity not only to Trolox but also to such flavonoids as dihydroquercetin (taxifolin) [20,24].

Due to the fact that the greatest mechanical loads on the knot tissues are created by the snow covering the tree branches, the conclusion was made and confirmed experimentally that the content of the lignans increases with the latitude at which the tree grows, as well as with the increase in its age [16,25]. This opens up serious prospects for the search for the most promising feedstock for obtaining lignans in subarctic territories with the most unfavorable climatic conditions. However, the available data on the chemical composition of knotwood extractives, their antioxidant properties, and the content of various lignans in the trees growing in this natural zone are geographically limited mainly to the territory of Finland and cannot be considered exhaustive. Moreover, even for the most studied spruce knotwood, various sources reported different or contradictory information. This may be due to both the high variability of the knotwood chemical composition, and the limitations associated with the applied analytical methodology based primarily on gas chromatography—mass spectrometry with the preliminary derivatization of the analytes and different approaches to the measurements of antioxidant activity.

The present study is aimed at a detailed characterization of the chemical composition and antioxidant activity of lignan-rich extractives obtained from the knotwood of four coniferous tree species growing near the northern border of their distribution area in the European North of Russia as a region with a highly developed forest industry. To achieve this, we propose a comprehensive approach based on a combination of the most powerful analytical techniques for non-targeted screening—two-dimensional NMR spectroscopy and high-performance liquid chromatography—high-resolution mass spectrometry (HPLC-HRMS) [26], and the determination of antioxidant activity using various complementary approaches.

## 2. Materials and Methods

### 2.1. Chemicals

Hexane, 1-butanol, and acetone (chem. pure) were purchased from Khimmed (Moscow, Russia), and type I ultra-pure Milli-Q water was used in the knotwood extraction procedures. Before use, acetone was additionally subjected to fractional distillation to remove the impurities.

In the analytical procedures, methanol (gradient HPLC grade), acetonitrile (high purity grade, Khimmed, Moscow, Russia, ethanol (pharm., 96%, KBZ, Kirov, Russia), and ortho-phosphoric acid (ACS reagent, ≥85%, Sigma-Aldrich, Steinheim, Germany) were used for the preparation of the mobile phase and sample solutions. DPPH (2,2-diphenyl-1-picrylhydrazyl radical), Gallic (97.5–102.5%), and ascorbic (puriss. p.a., ≥99.0%) acids, obtained from Sigma-Aldrich (Steinheim, Germany), and Trolox (analytical standard, Analytik Jena, Jena, Germany) were used in the antioxidant activity determination procedures. In NMR studies, deuterated dimethyl sulfoxide (DMSO-*d*_6_, ≥99.8%, Merck, Darmstadt, Germany) was used as a sample solvent.

### 2.2. Plant Material

As the objects of the study, the Scotch pine (*Pinus sylvestris*), Norway spruce (*Picea abies*), Siberian fir (*Abies sibirica*), and Larch (*Larix sibirica*) stemwood and knotwood samples were chosen due to the dominance of these tree species in the region and their role as an important raw material for the forest industry. The spruce and pine trees with the age of 60–70 and 40–50 years, respectively, were harvested in the north of the Arkhangelsk region between 64th and 65th degrees north latitude, while the fir and larch trees aged 40–50 and 80–90 years, respectively, were obtained from the southern part of the region at a latitude of 62° N (Supplementary Material Figure S1) close to the northern limit of the distribution of these tree species [27].

The parts of the trunk with large knots were cut from the five trees of each species at a height of ~3 m. The samples were drilled out from the stemwood and knotwood with a wood drill bit (diameter 3 cm) and were then combined before being carefully averaged for each species. The final weight of each average sample was about 1 kg. After 24 h vacuum drying at 40 °C, the obtained sawdust was additionally crushed in a ZM 200 centrifugal mill (Retsch, Haan, Germany) to a particle size of <1 mm.

### 2.3. Extraction Procedure

Isolation of the wood extractives was carried out using two common techniques—Soxhlet extraction and pressurized liquid extraction (PLE). The latter was chosen since it demonstrated high efficiency in the extraction of lignans when aqueous acetone (95%) was used as an extractant [13,28].

An exhaustive Soxhlet extraction was performed in an automatic extraction system B-811 (Buchi, Flawill, Switzerland). A 10 g portion of dry milled sawdust was placed in a cellulose thimble and extracted in two stages. At the first one, non-polar compounds (resin acids, fat, terpenes) were removed with hexane for 8 h. After air drying, at the second stage, the sample was also extracted with acetone for 8 h.

PLE was carried out using an ASE 350 accelerated solvent extraction system (Dionex, Sunnyvale, CA, USA). The sample (5 g) was placed in a stainless-steel extraction vessel with a volume of 22 mL and extracted in two stages, as in the case of the Soxhlet procedure using hexane and 95% aqueous acetone as extractants. Each stage consisted of three 15 min extraction cycles in a nitrogen atmosphere at a temperature of 100 °C and pressure of 100 bar. Between cycles, an extraction vessel was flushed with a fresh portion of the extractant (60% of the vessel volume). In additional experiments, butanol was used as an extractant at the second stage instead of 95% acetone, or the samples were extracted in the three stages (hexane, butanol, 95% aqueous acetone).

The obtained extracts were evaporated under a vacuum on a rotary evaporator to a volume of 5–7 mL and then mixed with an excess of water (100–150 mL). The obtained suspension of the extractives was immediately frozen with liquid nitrogen and then lyophilized in a FreeZone Triad freeze dryer (Labconco, Kansas City, MO, USA). After weighing, the dry extracts were stored in amber glass vials at 4 °C.

### 2.4. Antioxidant Activity Determination

An electrochemical (amperometric) determination of the antioxidant activity was carried out on a Blizar antioxidant analyzer (Interlab, Moscow, Russia) consisting of a chromatographic pump, a manual loop injector, and an electrochemical detector with a glassy carbon working and stainless-steel auxiliary (cell body) electrodes [29]. The sample was dissolved in a mixture of acetonitrile with water (1:1) containing 2.2 mM *ortho*-phosphoric acid, which was also used as a mobile phase. An analysis was performed by the flow injection of the sample solution (100 μL, 1 mg L^−1^) at a mobile phase flow rate of 1.2 mL min^−1^. The detection was carried out in a direct current mode with a working electrode potential of 1.3 V. Standard solutions of the gallic acid (0.2–4 mg L^−1^) were used for the calibration curve construction in the coordinates peak area—concentration. All measurements were performed in five replicates. The obtained RSD values for the antioxidant concentrations did not exceed 5%.

A DPPH free radical scavenging assay was carried out according to the known procedure [30]. The accurately weighed samples (~6 mg) were dissolved in 50 mL of ethanol and kept for 1 h in the dark. The sample solution (1 mL) was carefully mixed with 1 mL of ethanol and 2 mL of 0.3 mM DPPH ethanolic solution, then kept for 30 min in the dark. If required, the sample solution was diluted with ethanol to match the working concentration range. The blank sample was prepared in the same manner by replacing the sample solution with ethanol. Calibration solutions of the ascorbic acid in the ethanol were prepared in the concentration range of 10–30 mg L^−1^ immediately before the measurements. The absorbance was measured at a wavelength of 517 nm in 0.5 cm quartz cells on a Specord 250 Plus double-beam spectrophotometer (Analytik Jena AG, Jena, Germany). Antioxidant activity was calculated as a result of the three parallel measurements.

The superoxide anion radical scavenging activity was determined by the photochemiluminescence (PCL) technique using a Photochem analyzer (Analytik Jena AG, Jena, Germany) according to the known procedure [31]. The antioxidant capacity of the lipid-soluble (ACL) compounds determination protocol was used in combination with the commercially available ACL reagent kit (Analytik Jena AG, Jena, Germany). The calibration was performed immediately before the analysis using the Trolox standard solutions in methanol in the concentration range of 0.5–3 mg L^−1^. The sample solution in methanol (20 µL) with a concentration of 1 mg L^−1^ was mixed with ready-to-use ACL reagents according to the manufacturer’s manual and was introduced in the analyzer. All assays were performed in triplicate with further RSD calculation.

### 2.5. NMR Spectroscopy

About 30–50 mg of the dry extract was dissolved in 550 μL of DMSO-*d*_6_ and placed in the 5 mm (i.d.) NMR tubes. HSQC (heteronuclear single quantum correlation) and HMBC (heteronuclear multiple bond correlation) two-dimensional ^1^H-^13^C NMR spectra were recorded at 298 K on an AVANCE III 600 spectrometer (Bruker, Ettlingen, Germany) with an operating frequency for protons of 600 MHz and using sequences from the Bruker standard library. The specific experimental parameters are presented in Table 1. Bruker Topspin 3.2 software (Bruker, Ettlingen, Germany) was used for the registration and processing of the experimental data. The spectra were phased and calibrated to the solvent signal (δC/δH 39.5/2.5 ppm). The cross-peak assignment to identify the specific structures was performed by combining data from the HSQC and HMBC spectra using the ACD/Structure Elucidator expert system software (ACD/Labs, Toronto, ON, Canada), including an NMR spectral database (Supplementary Material Figure S2). The maximum allowable deviation for the protons did not exceed 1 ppm and 5 ppm for the carbon atoms.

### 2.6. HPLC-HRMS Analysis

Immediately before the analysis, the dry extracts were dissolved in a mixture of acetonitrile with water (1:1) and diluted with the same solvent to obtain a concentration of 500 mg L^−1^. The samples were filtered through a nylon membrane filter with a pore size of 0.2 µm and were introduced into the chromatograph.

The high-performance liquid chromatography—high-resolution mass spectrometry studies were carried out on an HPLC-HRMS system consisting of an LC-30 (Shimadzu, Kyoto, Japan) liquid chromatograph and a “tribrid” high-resolution mass spectrometer Orbitrap ID-X (Thermo Scientific, Waltham, MA, USA) with linear and orbital ion trap mass analyzers and an OptaMax NG ion source equipped with a heated electrospray (ESI) probe. The chromatography system consisted of two LC-30AD HPLC pumps, a DGU-5A vacuum degasser, an STO-30A column thermostat, an SIL-30AC autosampler, and an SPD-M20A diode array (DAD) UV-VIS spectrophotometric detector.

The chromatographic separation was carried out at 40 °C in a reverse-phase mode on a Nucleodur PFP column (Macherey-Nagel, Duren, Germany), 150 × 2 mm, with a pentafluorophenyl stationary phase (1.8 µm particle size). The mobile phase consisted of water (A) and acetonitrile (B), both containing 0.1% of formic acid. The flow rate was 0.25 mL min^–1^. The gradient elution was programmed as follows: 0–2 min—15% B, 2–25 min—linear ramp to 100% B, 25–30 min—100% B. The injection volume was 2.0 µL. A wavelength range of 200–500 nm, a spectral resolution of 4 nm, and an acquisition rate of 10 Hz were used in the spectrophotometric detection.

Mass spectrometry detection was carried out in a negative ion electrospray ionization mode (ESI(−)) with the following ion source parameters: spray voltage, 2.5 kV; sheath, auxiliary, and sweep gas (N_2_) flow rates—50, 10, and 2 arb. units, respectively; ion transfer tube and vaporizer temperature, 325 and 350 °C, respectively; S-lens RF level, 60 %. In the case of the Siberian fir extract analysis, an addition to the ESI(−) positive ion mode (ESI(+)) was also used with the same ion source parameters, excluding the spray voltage, which was set to 3.5 kV. The mass spectra were continuously acquired at an orbital ion trap resolving power of 120,000 (M/ΔM, at *m*/*z* 200) in the range of *m*/*z* 100–1000. The data-dependent acquisition of the tandem (MS/MS) mass spectra (dd-MS^2^ mode) with the precursor ion signal intensity threshold of 1.0 × 10^5^ cps as a criterion for quadrupole isolation and higher-energy collision-induced dissociation (HCD) was used in the non-target analysis. The HCD collision energy of 20 eV (collision gas—nitrogen) and a mass analyzer resolving power of 30,000 were applied. The mass scale calibration was performed on a daily basis using a Pierce standard mixture (Thermo Scientific, Waltham, MA, USA). To achieve the highest mass accuracy, an internal mass scale calibration (Easy-IC mode with fluoranthene as a standard) was used during the analysis. Xcalibur 4.4 software (Thermo Scientific, Waltham, MA, USA) was used to control the instrument and acquire HRMS chromatograms. Non-target screening and the identification of detected compounds were carried out using the Compound Discovery 3.3 software package (Thermo Scientific, Waltham, MA, USA) with an online search in the Chemspider and mzCloud databases. The following constraints were applied in the accurate mass-based elemental composition determination: the maximum allowed deviation of the measured *m*/*z* from the theoretical value—3 ppm, the maximum number of C, H, and O atoms—was 100, 200, and 50, respectively.

## 3. Results and Discussion

### 3.1. Exraction Efficiency and Yield of Extractives

The most important parameters that determine the economic efficiency of raw material processing are the content of the extractive substances and the possibility of their most complete extraction from plant tissues. In this regard, at the first stage of the study, both exhaustive Soxhlet extraction with acetone and PLE with 95% aqueous acetone showed high effectiveness [13,28,32] and were implemented into the treatment of the spruce knotwood sample previously deresined with hexane. In this experiment, practically the same extraction yields were observed for the Soxhlet and PLE techniques: 16 ± 2 and 14 ± 3%, respectively. Considering the identity of the chemical compositions of both extracts (according to 2D-NMR) and the tenfold higher rapidity of PLE along with the lower consumption of the extractant, only the latter extraction method was used in further research.

Since, from a practical point of view, “green” solvents have the greatest prospects as industrial extractants, butanol, currently considered one of the most available bio-based solvents and known for its ability to dissolve lignin-related substances, was chosen in our study as an alternative to acetone. However, the obtained results (Table 2) demonstrate that butanol, despite its higher basicity, is noticeably (by 20–30%) inferior in effectiveness to acetone in the extraction of knotwood. In the case of stemwood, which is much less rich in lignans, butanol provided a 2.5–3 times lower yield of extractives.

This can be explained by the lower polarity and an order of magnitude higher viscosity of butanol compared to acetone, which decreased the mass transfer during extraction. Nevertheless, butanol is still of interest as a lignan extractant, and extraction efficiency can probably be improved by increasing its duration or temperature. It is worth noting that the sequential extraction with butanol and aqueous acetone gives the same yields of extractives as a one-stage extraction with the latter solvent. This is additional evidence of achieving a complete extraction when using 95% acetone as the only extractant under the selected PLE conditions. The attained yield of extractives from the knotwood samples turned out to be ~5-fold higher compared to stemwood, and in the case of spruce, this difference reached 14 times.

A comparison of the yields of the extractives from the different knotwood samples showed significant variability—the minimum value (3.9%) was observed for Scotch pine, while Siberian pine knotwood gave 17% of the dry extract. In general, the found contents of the extractive substances in both the knot- and stemwood samples of coniferous tree species growing in the Russian North turned out to be close to the values presented in the literature for other regions (Finland, Slovenia) [12,32,33]. This casts doubt on the hypothesis about the dependence of lignan content on the geographical latitude of the tree growth and the snow cover height, mentioned in the introduction section. In any case, besides geographic latitude, there must be other abiotic and biotic factors influencing the formation of secondary metabolites in compression wood.

### 3.2. Antioxidant Activity of the Obtained Extracts

For a detailed characterization of the antioxidant activity of the obtained knotwood extracts, a combination of the three independent and complementary approaches, based on the single electron transfer (SET) and hydrogen atom transfer (HAT) reactions of antioxidants [34], were used: (i) amperometric method characterizing the ability to SET in the electrochemical processes; (ii) DPPH radical neutralization test revealing the ability to SET in a reaction with the organic radical the mechanism of which is similar to that with peroxyl radicals ROO^·^; (iii) photochemiluminescence (PCL) method characterizing the ability to quench superoxide anion radicals by HAT. The latter technique is based on the method developed by Popov and Lewin [35] and its close to the commonly used ORAC (oxygen radical absorbance capacity) approach.

**Figure 1:**
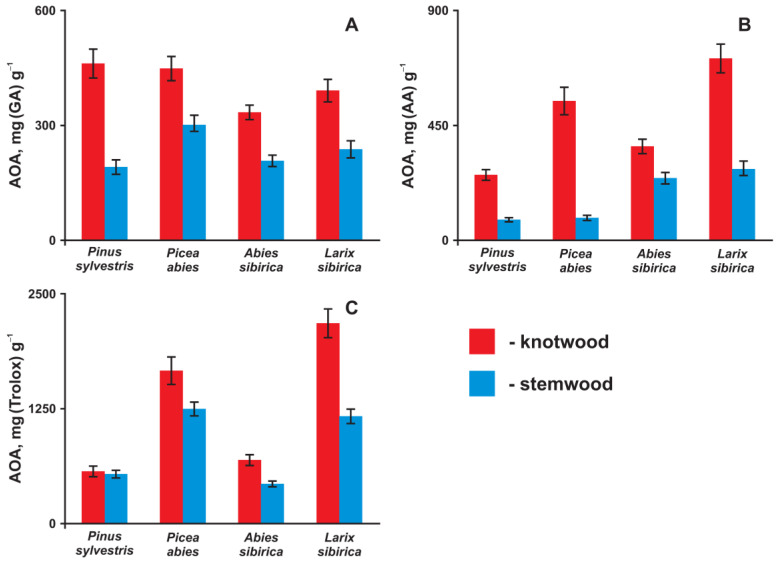
Antioxidant activity (AOA) of the knotwood and stemwood extractives determined in amperometric (**A**), DPPH (**B**), and PCL (**C**) assays. The AOA values are represented in gallic acid (GA), ascorbic acid (AA), and Trolox equivalents, respectively.

The obtained results (Figure 1) demonstrate the high antioxidant activity of all knotwood extracts, which was found to be 25–400% higher than that measured for the stemwood extractives. This is due to the known fact that the lignan content in knotwood extracts is much higher compared to that obtained from stemwood [23]. The absolute values of the antioxidant capacity fall in the ranges of 300–500, 250–720, and 550–2250 mg g^−1^ for amperometric (gallic acid units), DPPH (ascorbic acid units), and PCL (Trolox units) tests, respectively. Thus, it can be concluded that the knotwood acetone extracts are comparable in their antioxidant properties to the most well-known standards. Particular attention should be drawn to their exceptionally high ability to quench superoxide radicals since reactive oxygen species play a key role in the development of oxidative stress. In this parameter, the spruce and larch extractives turned out to be significantly superior (1.6- and 2.2-fold, respectively) to Trolox, which is in good agreement with the data obtained earlier in the lipid peroxidation tests [20].

Despite the different mechanisms underlying the used methods for determining antioxidant activity, the values obtained by the PCL and DPPH techniques correlate well with each other. As already mentioned, larch extractives have the highest antioxidant activity, and, according to this parameter, the obtained extracts can be arranged in the following order: *Pinus sylvestris* < *Abies sibirica* < *Picea abies* < *Larix sibirica.* However, the results obtained by the amperometric method are not related to the interaction with radical particles in the solution and stand somewhat apart. They are characterized by a completely different sequence in ascending order of the antioxidant properties: *Abies sibirica* < *Larix sibirica* < *Picea abies* < *Pinus sylvestris.* This phenomenon can be associated with a number of factors affecting electroactivity, including transport properties, adsorption on the electrode surface, etc. In any case, the observed variations in the antioxidant activity of the knotwood extractive substances indicate significant differences in the chemical composition of secondary metabolites synthesized by the studied tree species, the characterization of which was the subject of the studies described below.

### 3.3. NMR Screening of the Knotwood Extracts Chemical Composition

**Figure 2:**
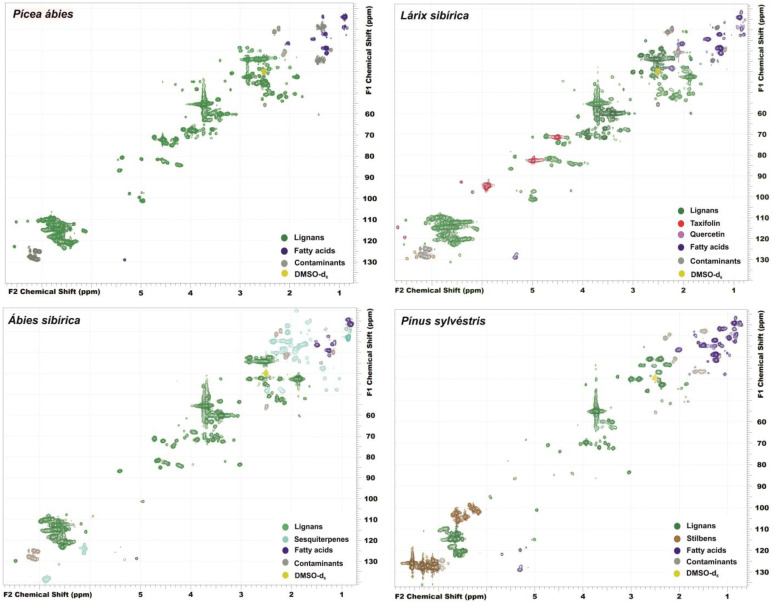
Two-dimensional (HSQC) ^1^H-^13^C NMR spectra of the *Picea abies*, *Pinus sylvestris*, *Abies sibirica*, and *Larix sibirica* knotwood extractives and cross-peaks assignment.

**Figure 3:**
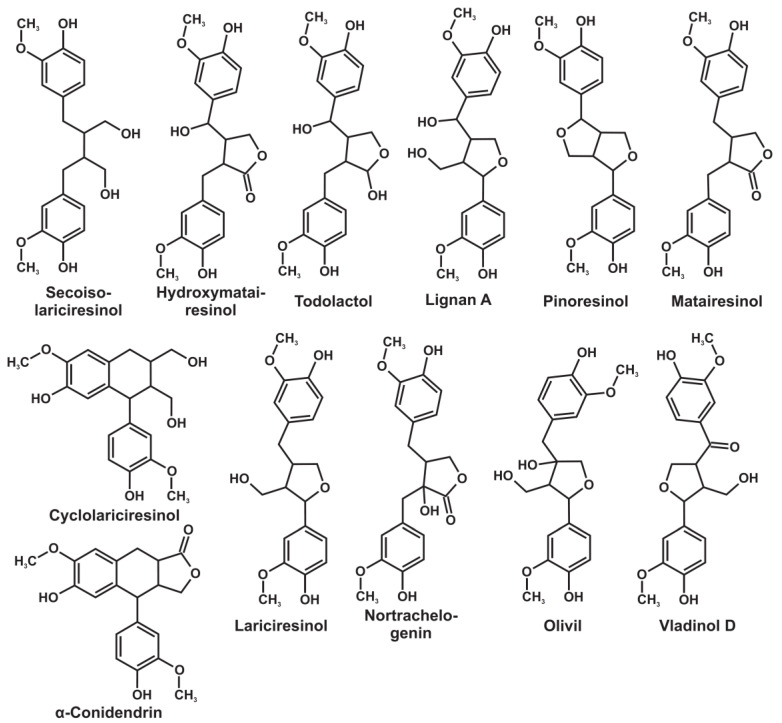
Structural formulas of the lignans identified in coniferous knotwood extracts.

The combination of high-resolution 2D-NMR with expert system software and database for analyzing the obtained spectra allows for the effective isolation of the sets of the unique cross-peaks of individual compounds and, thus, can be considered a powerful tool for the preliminary non-targeted screening of the chemical composition of plant secondary metabolites. The obtained spectra of the knotwood extractives (Figure 2) contain signals from several major groups of substances: lignans, flavonoids, fatty acids, stilbenes, terpenoids, residues of solvents and extractants, and unidentified compounds. A more detailed study of the NMR spectra and the tentative identification of the compounds belonging to the different groups (structural formulas are presented in Figure 3) allowed significant differences to be revealed in the chemical composition of polyphenolic compounds that dominate in the obtained knotwood extracts (Table 3).

Scotch pine knots not only contain the least amount of extractives, but also have the smallest variety of polyphenolic compounds. At the same time, lignans do not dominate among them; instead, stilbenes, which account for two-thirds of all aromatic structures, are detected by NMR. Major representatives of the latter group are pinosylvin and its methyl ether; small amounts of pterostilbene were also found. It should be noted that stilbenes were detected only in the *Pinus sylvestris* knotwood extract and were absent in other studied coniferous knotwood samples. Among the detected lignans, nortrachelogenin, also known as wikstromol, was found in the largest amounts accounting for 9% of all aromatic secondary metabolites and about 40% of the total lignan content, which is consistent with the literature data [12,36]. The group of minor lignans includes secoisolariciresinol and matairesinol (0.8% and 2.6% of the total aromatics content, respectively), as well as pinoresinol (0.2%) and todolactol (1.0%) which are not previously described in the literature for this object.

Siberian larch is another species in the knotwood extract of which, in addition to lignans, other polyphenolic compounds were found in significant amounts. An HSQC NMR spectrum of this sample contains intense signals of two flavonoids, quercetin and taxifolin, constituting 20% of all the aromatic structures. Unlike Scotch pine knotwood, the lignans in this sample are the main components and are much more diverse. This group is dominated by secoisolariciresinol (28% of the total aromatics content), followed by lariciresinol, todolactol, nortrachelogenin, lignan A, 5-hydroxymatairesinol and its isomers, pinoresinol, and vladinol D. Although major compounds listed above have already been previously identified in a larch knotwood [18,36], the latter four minor lignans were found in *Larix sibirica* knotwood for the first time.

As in the case of the Siberian larch, the dominant phenolic compounds of Siberian fir knotwood extractives are secoisolariciresinol and lariciresinol, which account for 44 and 15% of the total aromatics content, respectively. Among the known for this tree species’ lignans [21], 5-hydroxymatairesinol and its isomer, pinoresinol, and todolactol were found. The presence of lignan A in the *Abies sibirica* compression wood was not reported earlier; nevertheless, in the knotwood sample under study, the proportion of this secondary metabolite reached 4.7%, making it one of the major constituents. A feature of this sample also a presence of a certain amount of sesquiterpenes, the exact structures of which could not be established by NMR without the isolation of pure analytes.

The Norway spruce knotwood extract is distinguished by a strong predominance of 5-hydroxymatairesinol (HMR) in its chemical composition. This lignan (the sum of *cis-* and *trans*-isomers) constitutes 63% of all aromatic compounds, while the ratio of two detected isomers is close to 3:1. This observation is in good agreement with the results obtained by Willför et al. [13] for the spruce harvested in Finland. In addition to HMR, the studied sample contained secoisolariciresinol, α-conidendrin, lignan A, and lariciresinol with a sum content of ~12%, as well as two lignans (todolactol and vladinol D) which have not been previously found in the *Picea abies* knotwood.

The observed differences in the chemical composition of the knotwood extractive substances of various tree species are in good agreement with the data on the antioxidant activity of the obtained extracts (Figure 1). The anomalous position of the Scotch pine, which demonstrates the highest activity in the electrochemical test and the minimum values of antioxidant capacity in reactions with radical species (DPPH and superoxide anion radical), is explained by the strong domination of stilbenes (first of all, pinosylvin methyl ester) in its extract. As strong reducing agents due to the stabilization of the radical formed in the SET by a system of conjugated bonds, such compounds exhibit high electrochemical activity. At the same time, the low antioxidant capacity of pinosylvin methyl ester in reactions with radicals may be due to the presence of only one phenolic group in its structure.

Despite all the advantages of 2D NMR spectroscopy in establishing the structures of organic compounds, when analyzing such complex mixtures as plant extracts, there are a number of limitations in the interpretation of the spectra. This can explain the fact that several compounds, which account for 15–30% of the total aromatics content, remained unidentified in the samples under study. This problem can be solved by using liquid chromatography—high-resolution mass spectrometry as a complementary analytical technique, allowing the highly sensitive detection of the widest range of analytes without preliminary derivatization.

### 3.4. Analysis of the Knotwood Extracts by Liquid Chromatography—High-Resolution Mass Spectrometry

The non-targeted screening of the chemical composition of the obtained extracts made it possible to detect and establish the elemental compositions of 39 compounds belonging to the class of lignans, including 12 monomers (C_20_), and 27 oligomers with 30 (sesquilignans), 40 (dilignans), or 50 (sesterlignans) carbon atoms in their structure (Table 4).

Among the twelve identified lignan monomers possessing two aromatic rings (Figure 3), ten compounds were detected by 2D-NMR (Table 3). Thus, given that hydroxymatairesinol isomers have the same accurate mass, similar tandem mass spectra, and were not separated by HPLC (detected as one compound), the identification of all the lignans listed in Table 3 was confirmed by the independent mass spectrometry method based on elemental compositions and tandem mass spectra (Supplementary Material Figure S3) and can be considered reliable.

**Figure 4:**
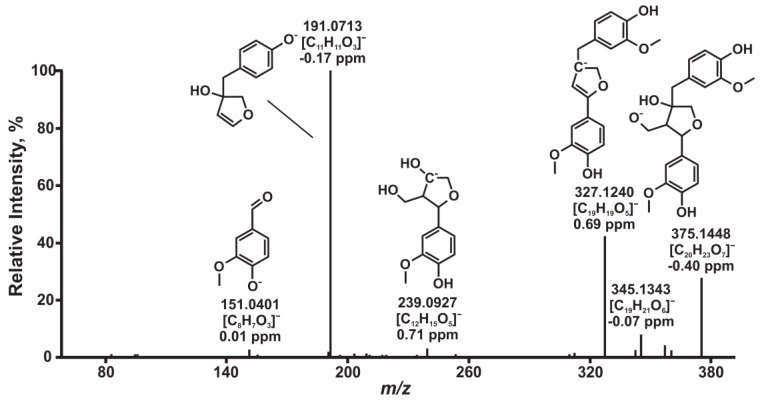
Tandem mass spectrum of the lignan C_20_H_24_O_7_ identified as olivil.

Two additional compounds in the group of monomeric lignans with elemental compositions of C_20_H_24_O_6_ and C_20_H_24_O_7_ were tentatively identified by HPLC-HRMS as cyclolariciresinol and olivil, respectively. The first compound was detected in trace amounts, and its identification was based primarily on the published data [23] obtained by GC-MS. On the contrary, olivil has not been previously found in the knotwood of the studied tree species despite the relatively high signal intensity and likely high content in all four samples. At the same time, another lignan with the same elemental composition, liovil, was described in the literature [23] as one of the major components of the fir knotwood extractives. The discrimination of the two isomers and confirmation of an olivil structure in the present study was attained using tandem mass spectrometry. The obtained MS/MS spectrum (Figure 4) contains the specific fragment ion with *m*/*z* 239.0927 and elemental composition C_12_H_15_O_5,_ which possesses two aliphatic hydroxy groups and is formed by the elimination of the methyl guaiacol moiety from the deprotonated molecule. Liovil, possessing both aliphatic OH groups at benzylic carbon atoms, cannot be fragmented by this pathway without the loss of the hydroxyl. This fact testifies in favor of the absence of liovil in the studied samples and the possibility of its erroneous identification in early studies by GC-MS. An alternative explanation suggests the co-elution of liovil and olivil due to the similarity of their structures and, thus, obtaining a mixed tandem mass spectrum.

An interesting observation is the detection of all the lignans listed in Table 4 in all the studied knotwood samples. This means that the differences between the coniferous tree species are not in the presence of specific compounds but in their ratios. This may be due to the biosynthesis in plant tissues of the same limited set of phenylpropane precursors, the combination of which with the formation of lignans can occur in a number of pathways with different probabilities. The latter depends on the conditions of lignan biosynthesis and determines the ratio of the resulting products.

This also applies to the more complex lignan structures (oligolignans), which were discovered to contain up to five phenylpropane units. This group of secondary metabolites is mainly represented by sesquilignans consisting of three phenylpropane units. The signal intensities of these compounds on the obtained HPLC-MS chromatograms are rather low, which may be due to the low ionization efficiency. An only exception is the knotwood of the Siberian fir, which is distinguished by the presence of intense peaks of dilignan C_40_H_50_O_12_ and sesquilignan C_30_H_36_O_9_, which are comparable to the signals of the most abundant monomeric compounds. It is worth noting that oligolignans were not detected by 2D-NMR since the corresponding signals overlapped with the cross-peaks of other compounds.

The reliable identification of their structures by mass spectrometry was also impossible, although a similar set of product ions in the tandem mass spectra of monomeric and oligomeric lignans indicated the similarity of their structures and their formation from the same initial phenylpropane precursors.

The identified non-lignan components of the Siberian larch knotwood extracts are expectedly represented by flavonoids quercetin (t_R_ = 10.4 min, *m*/*z* 301.0353, C_15_H_10_O_7_,) taxifolin (t_R_ = 7.2 min, *m*/*z* 303.0505, C_15_H_12_O_7_) and its methylated derivative (t_R_ = 8.9 min, *m*/*z* 317.0665, C_16_H_14_O_7_) and, presumably, naringenin (t_R_ = 11.2 min, *m*/*z* 271.0613, C_15_H_12_O_5_). The latter two compounds were found in low concentrations, so they were not identified by 2D-NMR. In the case of the Scotch pine, HPLC-HRMS analysis confirmed the presence of the three stilbene compounds detected by 2D-NMR—pinosylvin (t_R_ = 12.4 min, *m*/*z* 211.0764, C_14_H_12_O_2_), its methyl ether (t_R_ = 14.9 min, *m*/*z* 225.0922, C_15_H_14_O_2_), and pterostilbene (t_R_ = 14.9 min, *m*/*z* 255.1028, C_16_H_16_O_3_). The HPLC-HRMS chromatograms of the Norway spruce and Siberian fir knotwood extractives did not contain additional peaks of polyphenolic compounds. Nevertheless, of great interest is the identification of a group of abundant compounds in the Siberian fir extract, which according to the results of the 2D-NMR, was assigned to sesquiterpenes (Figure 2). Since these compounds have not been ionized by ESI(−), an additional experiment was performed in the positive ion mode. This allowed for the detection of the three compounds which were tentatively identified (by combining the HRMS and 2D-NMR data) as juvabione (t_R_ = 16.3 min, *m*/*z* 267.1955, C_16_H_26_O_3_) and its derivatives with one (t_R_ = 15.8 min, *m*/*z* 265.1795, C_16_H_24_O_3_) or two (t_R_ = 15.6 min, *m*/*z* 263.1637, C_16_H_22_O_3_) additional double bonds. The published data on the presence of juvabione in the tissues of trees of the *Abies* family [36,37,38] are an additional confirmation of the conclusions made.

Since ESI is highly susceptible to matrix interferences when studying samples of complex composition, to obtain more reliable quantitative estimates of component ratios, the combination of UV-DAD and mass spectrometric detection was used in the HPLC assays. Having the same chromophores (guaiacyl aromatic ring with λ_max_ ≈ 280 nm), lignans are characterized by their comparable absorption in the UV region; therefore, the obtained UV-DAD chromatograms (Supplementary Material Figure S4) give an adequate idea of the relative contents of the main polyphenolic components in the knotwood extracts. Generally, they confirm the results of the calculations based on 2D-NMR data. For example, the hydroxymatairesinol content in the Norway spruce knotwood extract, calculated based on HPLC-DAD chromatogram, was 68.5% which is very close to the value presented in Table 3 (65%). The same situation was observed for the nortrachelogenin, pinosylvin, and its methyl ether, the contents of which amounted to 6.5, 28, and 61%, respectively. A more detailed comparison of the quantitative estimates made by NMR and HPLC techniques was not possible due to the overlapping of the peaks of minor components in HPLC-DAD chromatograms, significant variations in the absorption coefficients between different classes of compounds, and large differences in the ionization efficiency during MS detection. Particular attention should be paid to the intensity of oligolignan signals on HPLC-DAD chromatograms, which generally correspond to the proportion of aromatic compounds not identified by 2D-NMR. Thus, the question of their nature can be considered resolved.

## 4. Conclusions

The knotwood of coniferous trees growing in the European North of Russia contains between 3.9 (Scotch pine) and 17% (Norway spruce) extractive substances with high antioxidant activity both in the single-electron transfer processes (amperometric and DPPH tests) and quenching of superoxide radicals by the hydrogen atom transfer mechanism (photochemiluminescent assay), in some cases surpassing Trolox in this parameter. The high antioxidant capacity of the obtained aqueous acetone extracts is due to the predominance of polyphenolic compounds, primarily lignans. The latter are represented by 12 main compounds, including several lignans identified for the first time in the knotwood of the studied coniferous tree species, and 27 oligolignans, containing between three and five phenylpropane units in their structure. An important established fact is that the obtained extracts are characterized by an identical set of lignans and differ only in the ratios of individual components, which indicates the commonality of the precursors and pathways for the biosynthesis of such compounds in coniferous trees. Other polyphenolic components of the knotwood are flavonoids taxifolin and quercetin (Siberian larch) and three stilbene compounds (pinosylvin, its methyl ester, and pterostilbene), which are major components of the Scotch pine extractives. Of the non-aromatic compounds, sesquiterpene juvabione and its derivatives were found in extracts of the Siberian larch knotwood. Further research aimed at the valorization of coniferous knotwood while obtaining individual compounds with antioxidant properties and other types of bioactivities should be focused on the development of the methods for the efficient preparative chromatographic separation and purification of the identified lignans.

## Figures and Tables

**Table 1:** 2D-NMR spectra registration parameters.

Parameter	Experiment	
	^1^H-^13^C HSQC	^1^H-^13^C HMBC
Pulse sequence	hsqcedetgpsisp2.3	hmbcetgpl3nd
Number of points	1024 × 256	2048 × 512
Number of scans	4	8
Spectral window width, ppm	12/240	10/240
Delay, sec	4	2

**Table 2:** Yield of stemwood and knotwood extractives (calculated on oven-dry wood material) obtained by the pressurized liquid extraction (100 °C, 100 bar) with different extractants.

Tree Species	Wood Type	Yield, %		
		Acetone *	Butanol	Acetone * (After Butanol)
*Pinus sylvestris*	stemwood	0.74 ± 0.10	0.27 ± 0.05	0.54 ± 0.06
	knotwood	3.9 ± 0.5	2.7 ± 0.3	1.2 ± 0.1
*Picea abies*	stemwood	1.0 ± 0.2	0.3 ± 0.04	0.7 ± 0.1
	knotwood	14 ± 3	11 ± 2	3.8 ± 0.4
*Abies sibirica*	stemwood	3.0 ± 0.4	1.2 ± 0.2	2.0 ± 0.2
	knotwood	17 ± 3	13 ± 3	5.6 ± 0.7
*Larix sibirica*	stemwood	1.9 ± 0.2	0.62 ± 0.1	1.3 ± 0.2
	knotwood	11 ± 2	8.3 ± 0.9	3.3 ± 0.4

* With the addition of 5% water.

**Table 3:** Polyphenolic compounds tentatively identified in the knotwood extracts by 2D-NMR and their relative contents (%, of the total aromatics content).

Group	Compound	*Pinus* *sylvestris*	*Picea* *abies*	*Abies* *sibirica*	*Larix* *sibirica*
Lignans	Nortrachelogenin	9.1	-	-	2.9
	5-Hydroxymatairesinol	-	46	6.5	-
	5-Hydroxymatairesinol (*cis-trans* isomer)	-	17	1.6	2.1
	Todolactol	1.0	5.1	0.7	3.4
	Secoisolariciresinol	0.8	4.8	44	28
	*iso*-Hydroxymatairesinol	-	3.0	-	1.6
	α-conidendrin	-	2.4	-	-
	Pinoresinol	0.2	-	1.0	0.4
	Lariciresinol	-	1.4	15	9.3
	Vladinol D	-	0.8	-	0.4
	Matairesinol	2.6	0.9	-	-
	Lignan A	-	3.5	4.7	2.6
Flavonoids	Taxifolin	-	-	-	19
	Quercetin	-	-	-	1.0
Stilbenes	Pinosylvin	20	-	-	-
	Pinosylvin methyl ether	45	-	-	-
	Pterostilbene	2.1	-	-	-
	Total	80.8	84.9	73.5	70.7

**Table 4:** Lignans detected in coniferous knotwood extracts by HPLC-HRMS.

Compound	t_R_ *, min	*m*/*z* [M–H]^−^	Elemental Composition	Δ*m*/*z*, ppm	Peak Area, ×10^7^, Arb. Units			
					*Pinus* *sylvestris*	*Picea* *abies*	*Abies* *sibirica*	*Larix* *sibirica*
			monomers					
Lignan A **	5.2	375.1448	C_20_H_24_O_7_	−0.32	0.3	1.5	2.2	0.4
Todolactol **	6.2	375.1447	C_20_H_24_O_7_	−0.56	0.4	1.9	0.5	1.7
Olivil	7.4	375.1448	C_20_H_24_O_7_	−0.40	2.1	8.2	1.2	4.3
Hydroxymatairesinol **	8.4	373.1286	C_20_H_22_O_7_	−1.68	1.3	177	29.5	5.5
Secoisolariciresinol **	8.5	361.1652	C_20_H_26_O_6_	−1.1	0.6	1.5	15.0	11.6
Lariciresinol **	8.5	359.1503	C_20_H_24_O_6_	0.57	0.01	0.01	0.1	0.07
Vladinol D **	8.6	373.1290	C_20_H_22_O_7_	−0.73	1.3	0.1	1.4	2.5
Cyclolariciresinol	9.0	359.1499	C_20_H_24_O_6_	−0.39	<0.01	<0.01	0.08	0.05
Pinoresinol **	9.6	357.1342	C_20_H_22_O_6_	−0.58	<0.01	0.01	0.3	0.02
Nortrachelogenin **	9.8	373.1289	C_20_H_22_O_7_	−0.99	29.7	0.2	0.08	5.0
α−Conidendrin **	10.8	355.1186	C_20_H_20_O_6_	−0.42	0.01	0.6	0.02	0.02
Matairesinol **	11.3	357.1338	C_20_H_22_O_6_	−1.34	9.1	4.3	1.7	1.1
			oligomers					
Sesquilignan	6.7	571.2183	C_30_H_36_O_11_	−0.39	0.04	0.3	0.4	0.05
Sesquilignan	7.3	571.2182	C_30_H_36_O_11_	−0.50	0.2	1.2	0.5	0.1
Sesquilignan	7.6	571.2184	C_30_H_36_O_11_	−0.07	0.04	0.1	0.4	0.05
Sesquilignan	8.1	571.2183	C_30_H_36_O_11_	−0.31	1.3	4.1	1.0	1.1
Sesquilignan	8.7	569.2026	C_30_H_34_O_11_	−0.46	0.04	0.4	0.4	0.04
Sesquilignan	8.9	569.2024	C_30_H_34_O_11_	−0.77	0.06	3.5	0.7	0.07
Sesquilignan	8.9	555.2234	C_30_H_36_O_10_	−0.22	0.03	0.3	0.8	0.5
Sesquilignan	9.0	555.2232	C_30_H_36_O_10_	−0.72	0.07	2.4	4.2	2.3
Sesquilignan	9.2	555.2233	C_30_H_36_O_10_	−0.44	0.01	0.7	1.9	0.6
Sesquilignan	9.4	553.2082	C_30_H_34_O_10_	0.56	0.4	2.4	0.6	0.3
Sesquilignan	9.5	569.2029	C_30_H_34_O_11_	0.08	0.5	<0.01	<0.01	0.07
Sesquilignan	9.6	569.2026	C_30_H_34_O_11_	−0.46	1.9	0.1	<0.01	0.2
Sesquilignan	10.1	539.2286	C_30_H_36_O_9_	−0.11	0.2	0.7	10.2	1.8
Sesquilignan	10.3	551.1924	C_30_H_32_O_9_	0.14	0.06	5.2	0.9	0.05
Dilignan	10.5	721.3222	C_40_H_50_O_12_	−1.03	0.07	0.2	22.9	2.2
Dilignan	10.7	719.3069	C_40_H_48_O_12_	−0.49	<0.01	0.03	2.0	0.4
Dilignan	10.7	733.2862	C_40_H_46_O_13_	−0.51	0.01	1.5	4.8	0.03
Sesquilignan	10.9	553.2082	C_30_H_34_O_10_	0.56	1.0	0.04	0.07	0.07
Dilignan	10.9	719.3071	C_40_H_48_O_12_	−0.32	0.01	0.03	3.2	0.4
Sesquilignan	11.1	551.1926	C_30_H_32_O_9_	0.65	1.0	0.03	<0.01	0.09
Sesterlignan	11.1	915.3809	C_50_H_60_O_16_	0.05	<0.01	0.2	0.3	0.6
Sesterlignan	11.1	899.3856	C_50_H_60_O_15_	−0.36	<0.01	<0.01	0.2	0.6
Dilignan	11.2	719.3069	C_40_H_48_O_12_	−0.62	<0.01	0.9	1.2	3.2
Sesquilignan	11.6	535.1976	C_30_H_32_O_9_	0.52	0.02	0.05	0.08	0.06
Sesquilignan	12.1	535.1976	C_30_H_32_O_9_	0.40	0.7	0.05	0.05	0.05
Dilignan	12.4	717.2915	C_40_H_46_O_12_	−0.20	0.01	1.3	0.4	0.3
Sesquilignan	13.6	535.1977	C_30_H_32_O_9_	0.63	<0.01	<0.01	0.9	0.1

*—retention time; **—also detected by 2D-NMR.

## Data Availability

The data presented in this study are available in the article and Supplementary Materials.

## Supplementary Materials

The following supporting information can be downloaded at: https://www.mdpi.com/article/10.3390/antiox11122338/s1, Figure S1: Areas of tree harvesting in the Arkhangelsk region of Russia; Figure S2: An example of 2D-NMR workflow for unknown compound structure elucidation in complex mixture using ACD/Labs expert system; Figure S3: Tandem mass spectra of the major lignans in knotwood extracts and assignment of the product ions (A—hydroxymatairesinol; B—secoisolariciresinol; C—nortrachelogenin); Figure S4: HPLC-DAD chromatograms (280 nm) of the knotwood PLE extracts.
