# Supplementary material for: Comprehensive Characterization of Chemical Composition and Antioxidant Activity of Lignan-Rich Coniferous Knotwood Extractives
Source: Antioxidants (Basel). 2022 Nov 25;11(12):2338. doi: 10.3390/antiox11122338 (PMC9774788; doi:10.3390/antiox11122338)
Supplement: Supplementary file 1 [file antioxidants-11-02338-s001.zip › antioxidants-2048493-supplementary.pdf]

Supplementary material

Comprehensive Characterization of Chemical Composition and Antioxidant Activity of Lignan-rich Coniferous Knotwood Extractives

Contents:

Figure S1. Areas of trees harvesting in the Arkhangelsk region of Russia.

Figure S2. An example of 2D-NMR workflow for unknown compound structure elucidation in complex mixture using ACD/Labs expert system.

Figure S3. Tandem mass spectra of the major lignans in knotwood extracts and assignment of the product ions (A – hydroxymatairesinol; B – secoisolariciresinol; C – nortrachelogenin).

Figure S4. HPLC-DAD chromatograms (280 nm) of the knotwood PLE extracts.

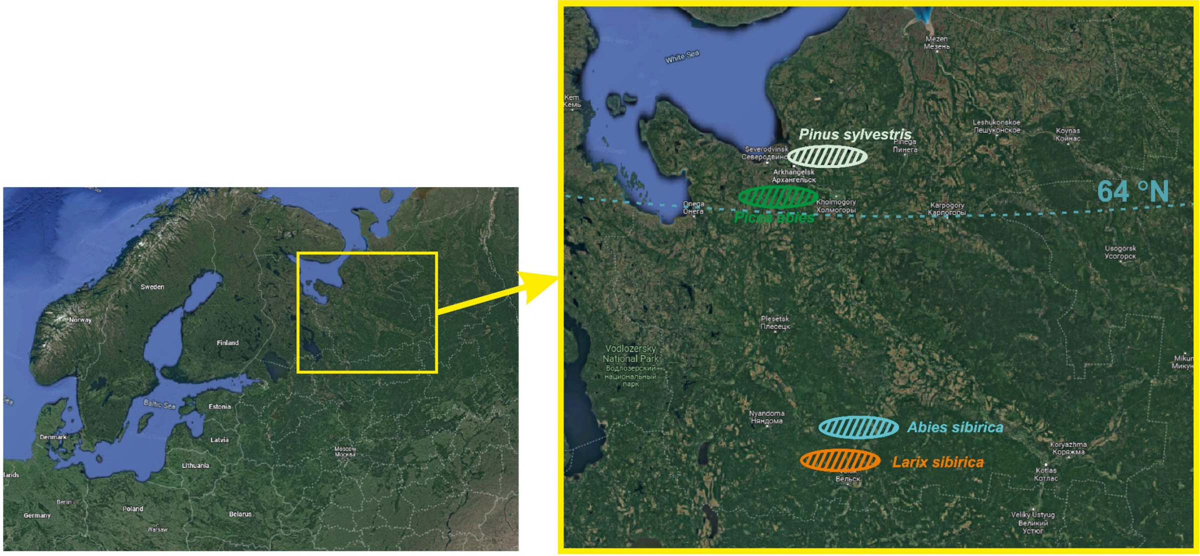

Figure S1. Areas of trees harvesting in the Arkhangelsk region of Russia

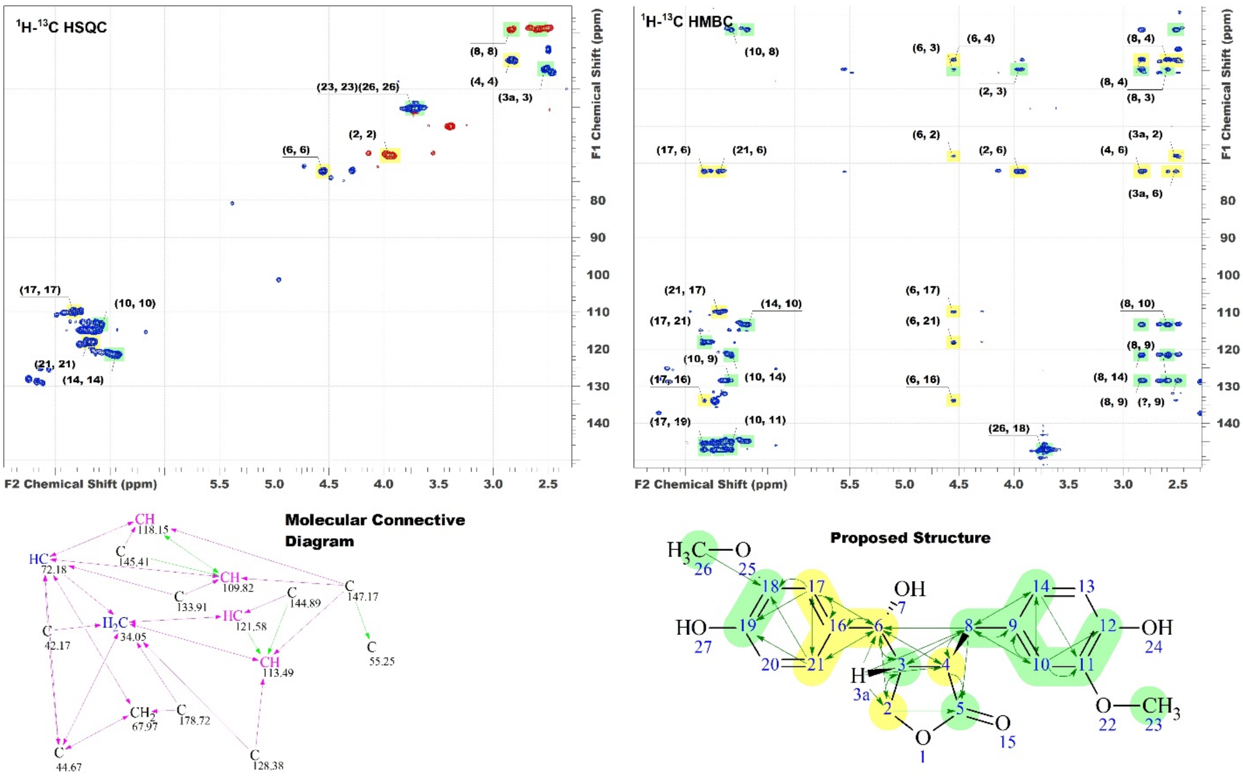

Figure S2. An example of 2D-NMR workflow for unknown compound structure elucidation in complex mixture using ACD/Labs expert system

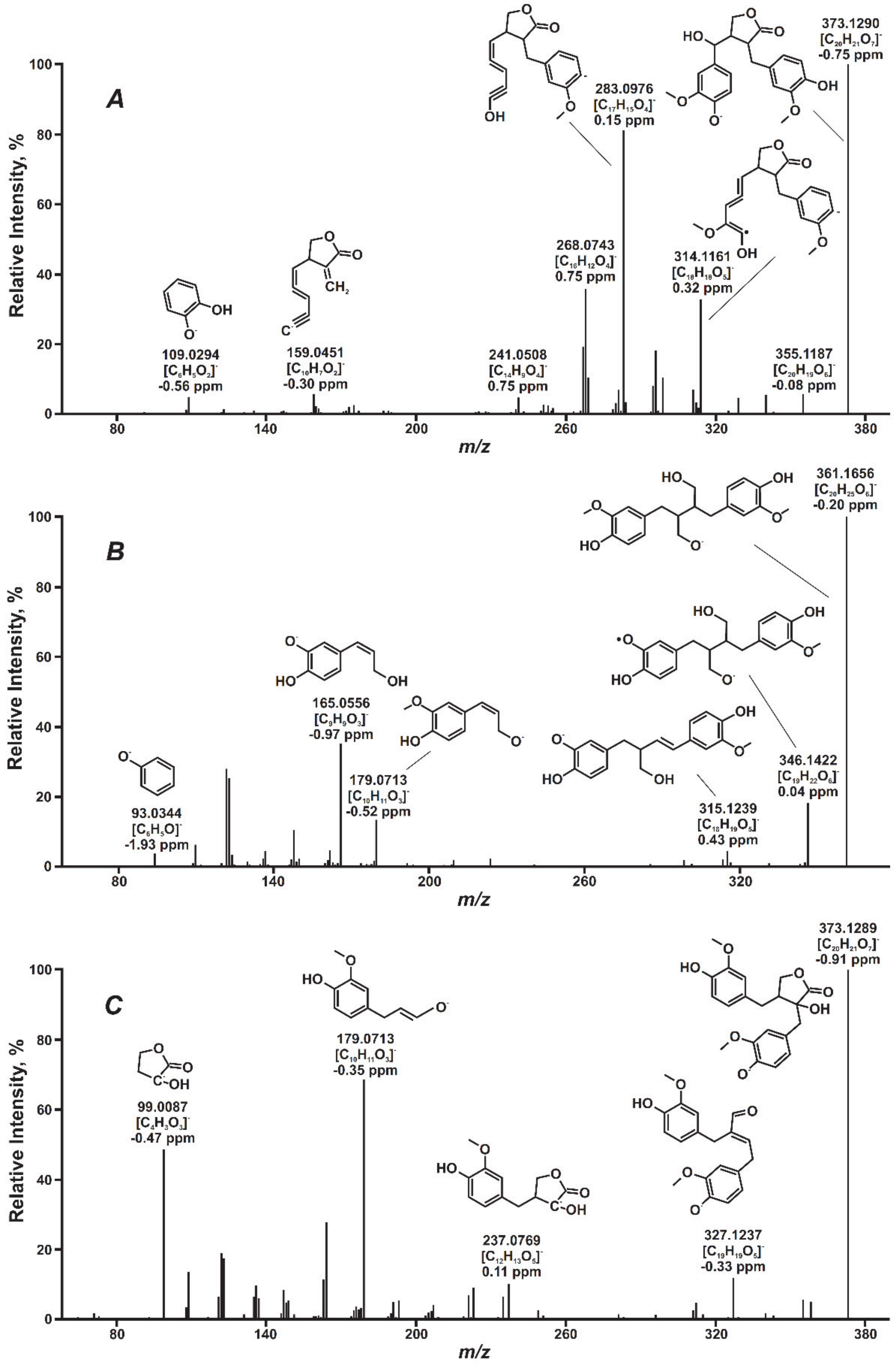

Figure S3. Tandem mass spectra of the major lignans in knotwood extracts and assignment of the product ions (A – hydroxymatairesinol; B – secoisolariciresinol; C – nortrachelogenin)

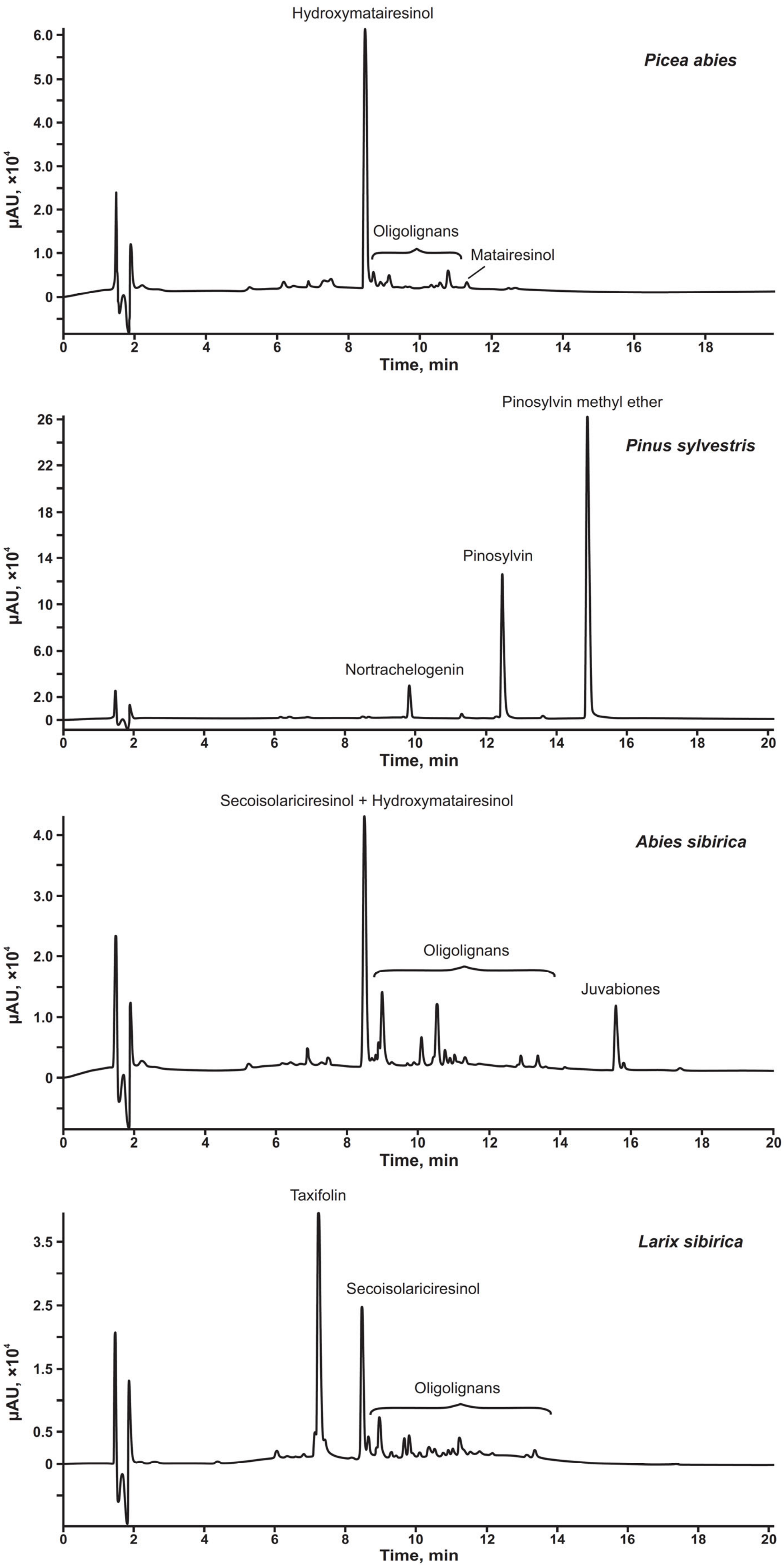

Figure S4. HPLC-DAD chromatograms (280 nm) of the knotwood PLE extracts
